# Supplementary figures and images for: EGFRAP encodes a new negative regulator of the EGFR acting in both normal and oncogenic EGFR/Ras-driven tissue morphogenesis
Source: PLoS Genet. 2021 Aug 19;17(8):e1009738. doi: 10.1371/journal.pgen.1009738 (PMC8407591; doi:10.1371/journal.pgen.1009738)

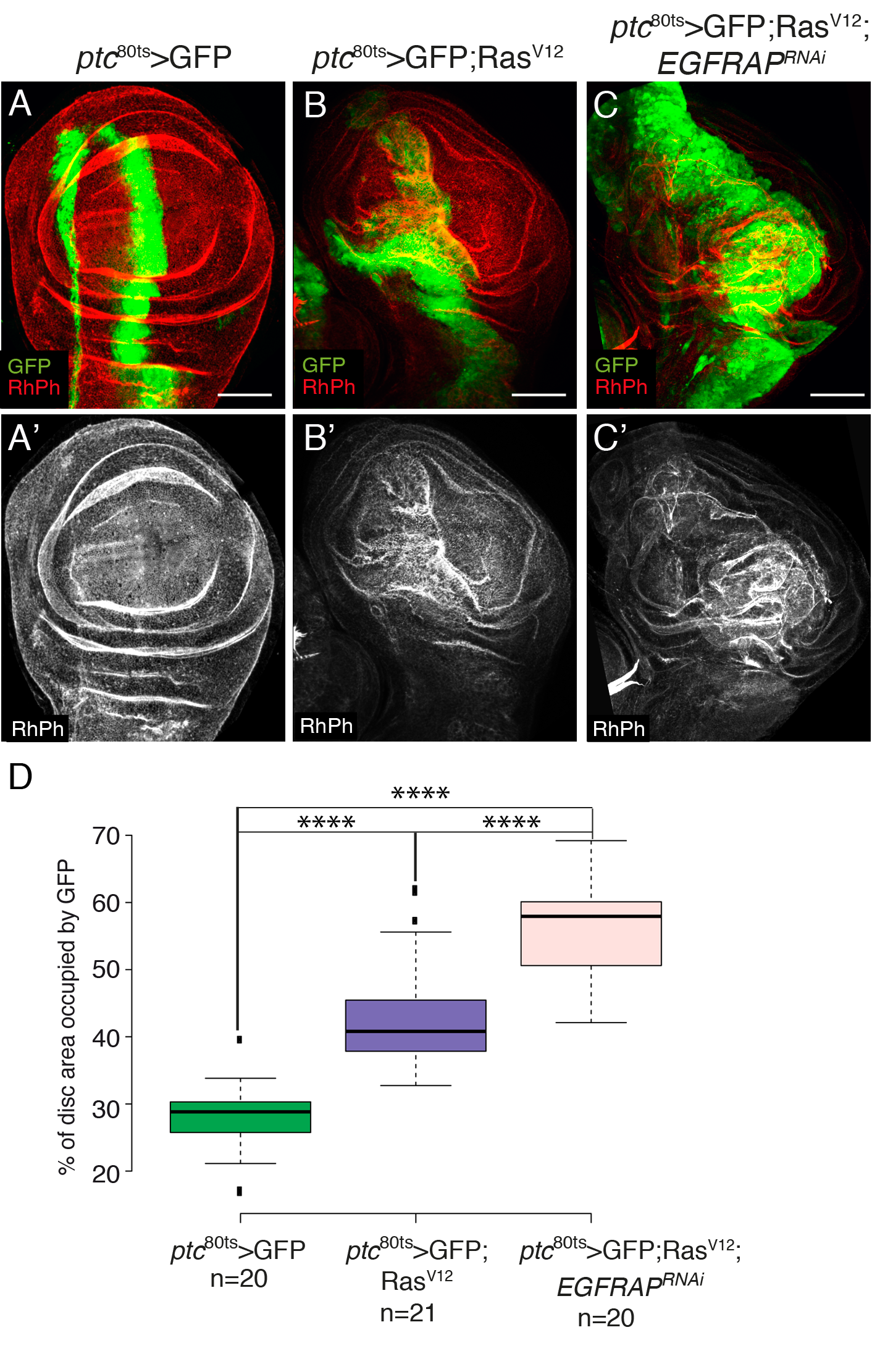

Supplement: S1 Fig — (A-C’) Maximal projection of confocal images of wing imaginal discs from third-instar larvae expressing the indicated UAS transgenes under the control of ptc80ts-Gal4 line stained with anti-GFP (green) and RhPh (red). (D) Box plot of the area of the wing disc occupied by GFP+ cells of the indicated genotypes. The statistical significance of differences was assessed with a t-test, ****P value<0.0001. Scale bars, 50 μm (A-C). (TIF) [file pgen.1009738.s001.tif]

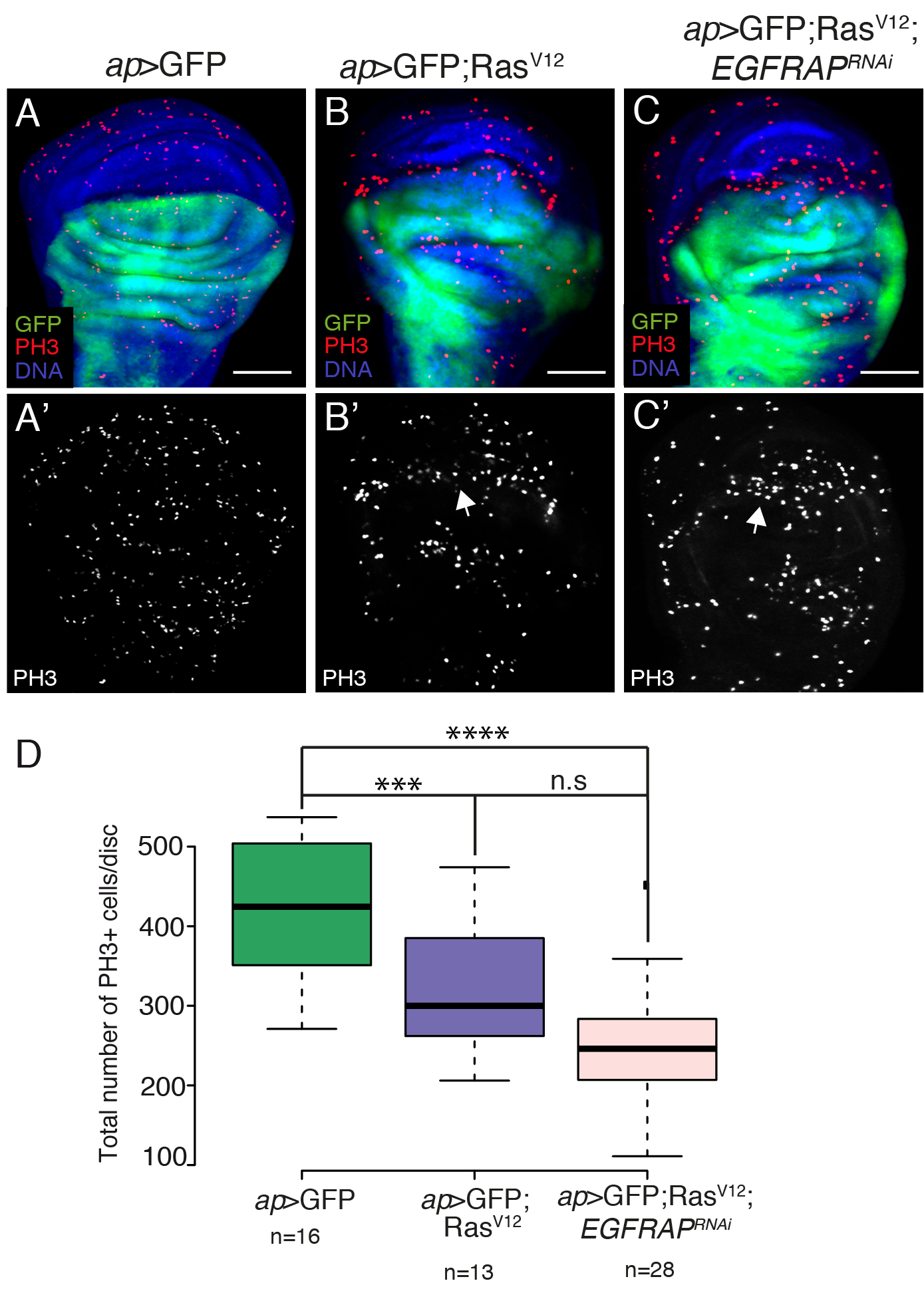

Supplement: S2 Fig — (A-C’) Maximal projection of confocal images of wing imaginal discs from third-instar larvae expressing the indicated UAS transgenes under the control of ap-Gal4, stained with anti-GFP (green), anti-Phosphohistone 3 (PH3, red) and Hoechst (DNA, blue). (D) Box plots of total number of PH3+ cells/disc of the designated genotypes. The statistical significance of differences was assessed with a t-test, **** and *** P values <0.0001 and <0.001, respectively. Scale bars, 50 μm (A-C). (TIF) [file pgen.1009738.s002.tif]

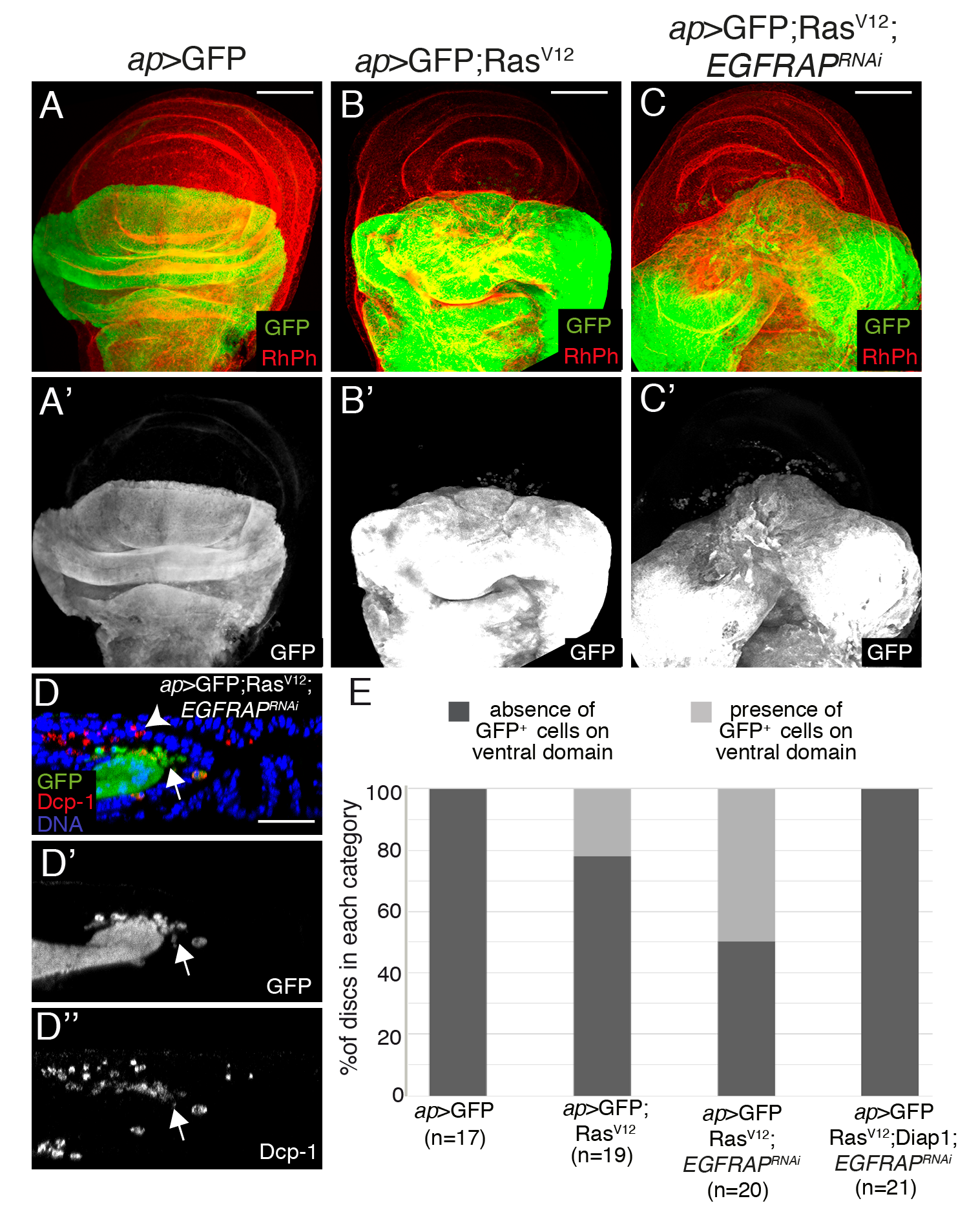

Supplement: S3 Fig — (A-C’) Maximal projection of confocal images of wing imaginal discs from third-instar larvae expressing the indicated UAS transgenes under the control of ap-Gal4, stained with anti-GFP (green) and RhPh (red). (D-D”) Confocal cross-sections of an ap>GFP; RasV12; EGFRAPRNAi wing disc stained anti-GFP (green), anti-Dcp1 (red) and Hoechst (DNA, blue). White arrows point to dorsal tumor cells (GFP+) invading the ventral compartment. White arrowheads point to control ventral cells (GFP-) undergoing apoptosis. (E) Quantification of wing discs of the indicated genotypes with (pale grey) or without (strong grey) dorsal tumor cells (GFP+) in the ventral compartment. Scale bars, 50 μm (A-C) and 30 μm (D-D”). (TIF) [file pgen.1009738.s003.tif]

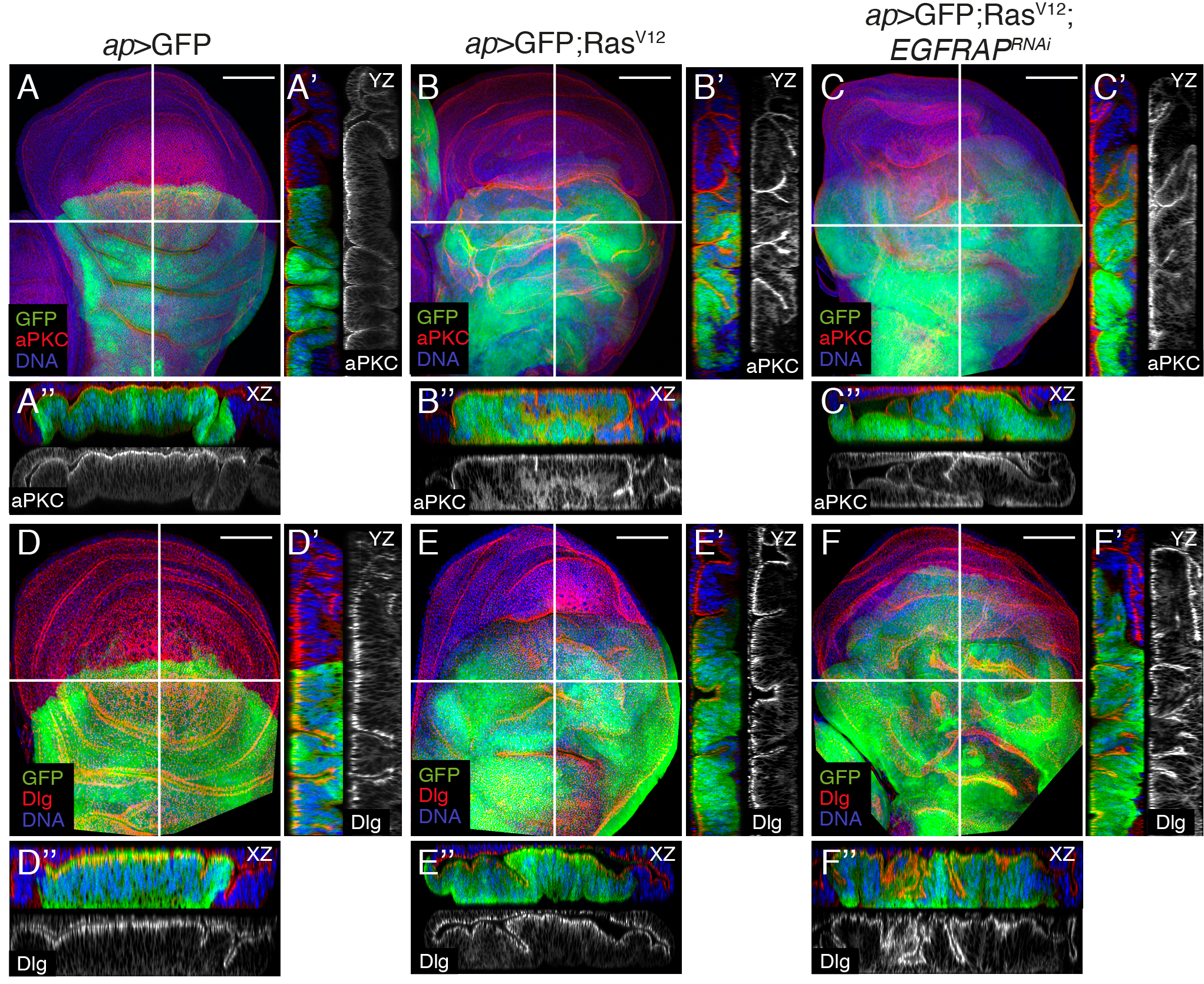

Supplement: S4 Fig — (A-F) Maximal projection of confocal views of third instar wing imaginal discs expressing the indicated UAS transgenes under the control of ap-Gal4, stained with anti-GFP (green), anti-aPKC (A-C, red) or anti-Dlg (D-F, red) and Hoechst (DNA, blue). (A’-A”-F’-F”) Confocal sections of wing discs of the indicated genotypes along the white dotted lines shown in A-F, respectively, parallel (A’-F’) or perpendicular (A”-F”) to the A/P axis. Apical sides of wing discs are to the left (A’-F’) or top (A”-F”). Scale bars, 50 μm (A-F). (TIF) [file pgen.1009738.s004.tif]

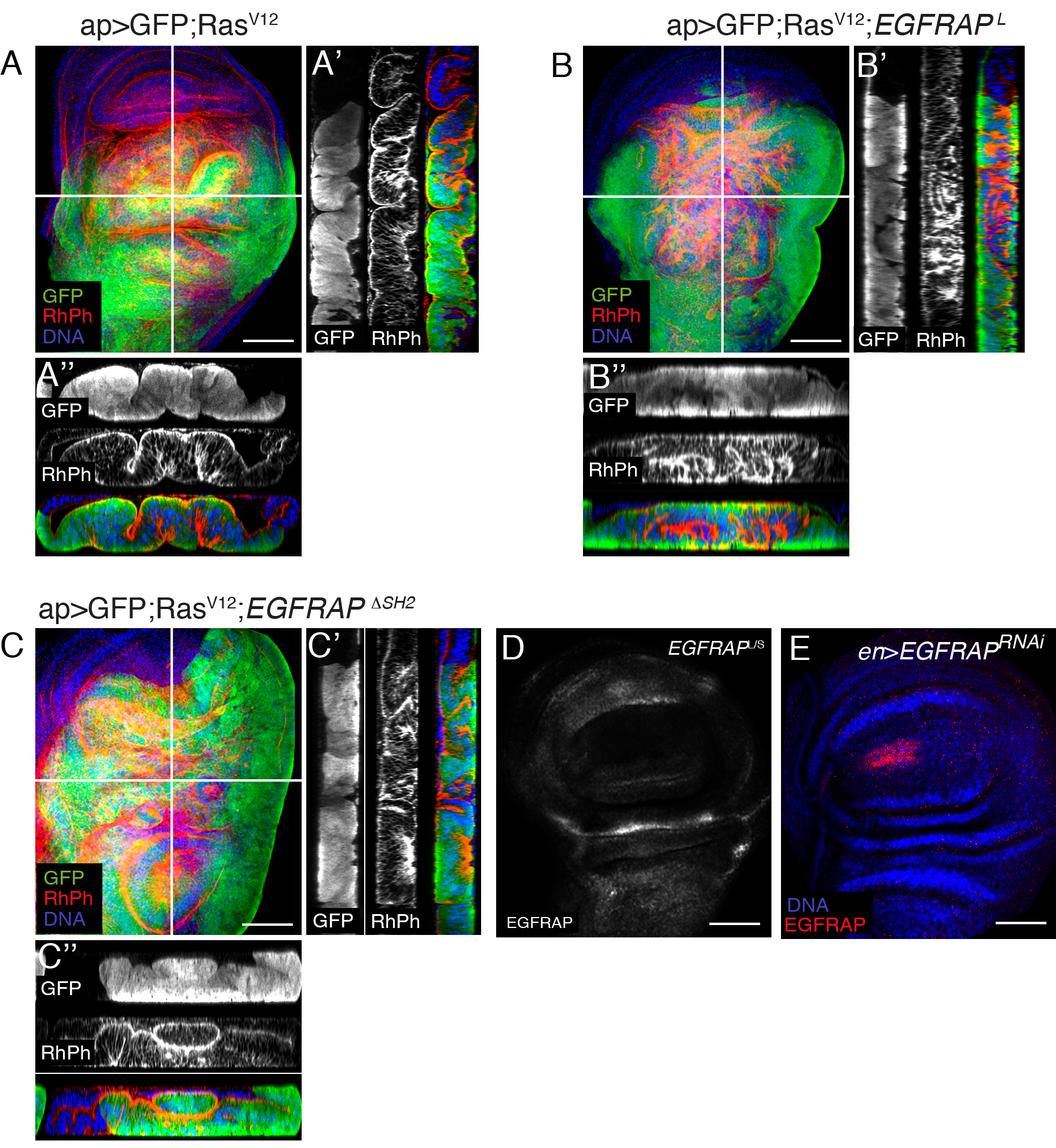

Supplement: S5 Fig — (A-C) Maximal projection of confocal views of third instar wing imaginal discs of the indicated genotypes, stained with anti-GFP (green), RhPh (red) and Hoechst (DNA, blue). (A’-A”-C’-C”) Confocal sections of wing discs of the indicated genotypes along the white lines shown in A-C, respectively, parallel (A’, B’ and C’) or perpendicular (A”, B”, and C”) to the A/P axis. Apical sides of wing discs are to the left (A’, B’ and C’) or top (A”, B” and C”). (D) EGFRAPL/S mutant wing disc stained with anti-EGFRAP. (E) Wing disc expressing a EGFRAP RNAi with en-Gal4, stained with anti-EGFRAP (red) and Hoechst (DNA, blue). Scale bars, 50 μm (A-E). (TIF) [file pgen.1009738.s005.tif]

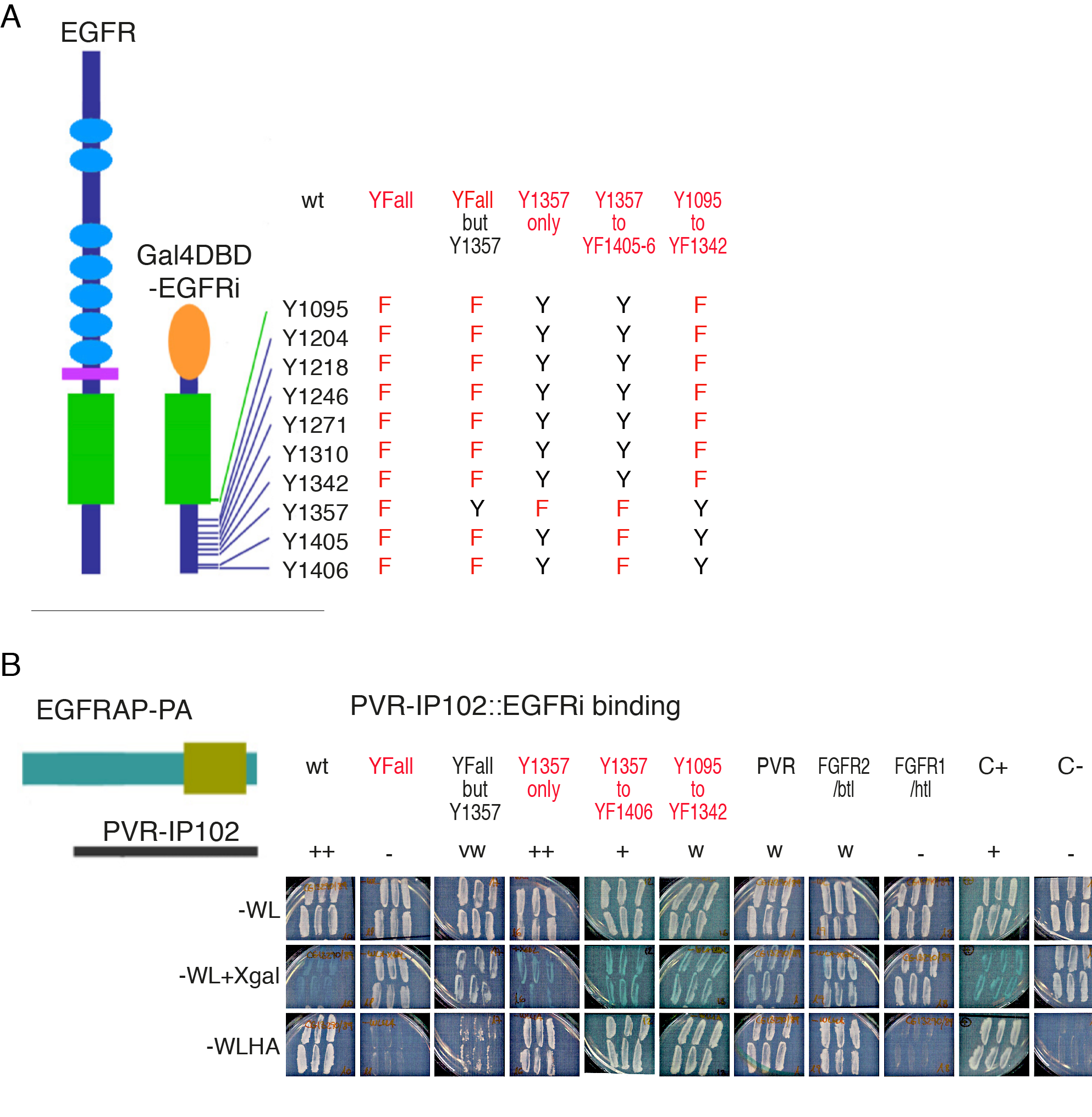

Supplement: S6 Fig — (A) Scheme of EGFR, the different baits used in the yeast two-hybrid assays and the tyrosine to phenylalanine mutations introduced in the cytoplasmic domain of EGFR (EGFRi). (B) Left: scheme of the EGFRAP-PA protein, and probable length of the partial clone PVR-IP#102 (black bar). Right: Interactions detected in yeast cells co-transformed with the PVR-IP#102 prey and different baits. -WL, medium lacking Trp and Leu. -WL+Xgal, medium lacking Trp and Leu supplemented with Xgal substrate. -WLHA, medium lacking Trp, Leu, His and Adenine. Interactions between candidate proteins were assessed by analyzing both β-galactosidase activity (-WL+Xgal) and the ability to grow in the absence of His and Adenine (-WLHA): ++ very strong interaction; + strong interaction: w, weak interaction; vw, very weak interaction;—no interaction; relative to the positive control. C+, positive control; C-, negative control. (TIF) [file pgen.1009738.s006.tif]

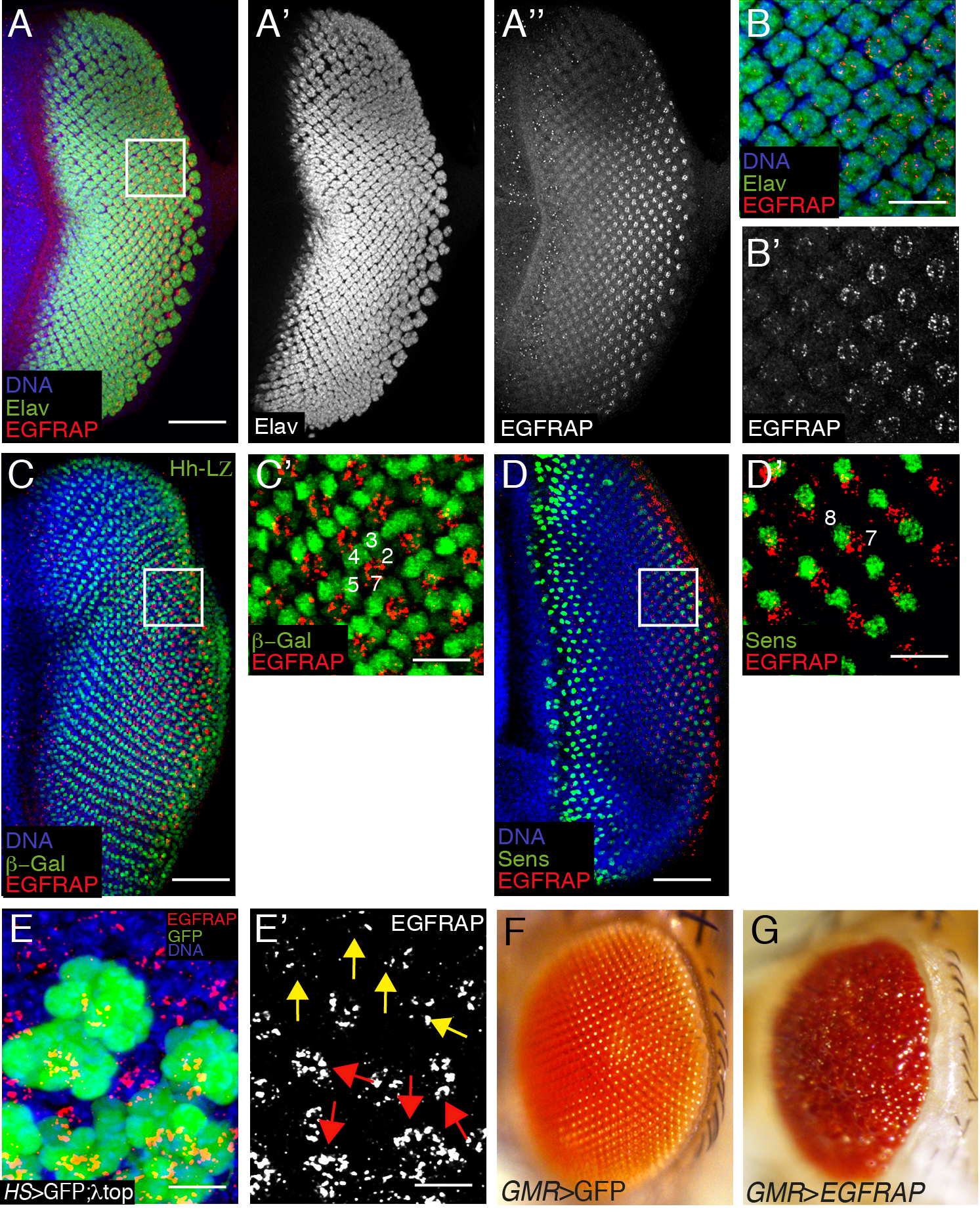

Supplement: S7 Fig — (A-D’) Confocal views of wild-type (A-B’ and D, D’) and Hedgehog-LacZ (Hh-LZ, C, C’) eye imaginal discs stained with anti-Elav (green in and B, white in A’), anti-EGFRAP (red in A, C, C’, D and D’ and white in A” and B’), anti-βgal (green in C, C’), anti-Senseless (Sens, green in D, D’) and Hoechst (DNA, blue, A, B, C, D and E). B-B’, C’ and D’ are magnifications of the white boxes in A, C and D, respectively. (E, E’) Eye discs carrying small clones of λtop expressing cells (GFP+) stained with anti-GFP (green), anti-EGFRAP (red in E, white in E’) and Hoechst (DNA, blue). (F, G) Adult female Drosophila eyes of the indicated genotypes. Scale bars, 50 μm (A-A”, C, D); 10 μm (B, B’, C’, D’), 5 μm (E, E’). (TIF) [file pgen.1009738.s007.tif]

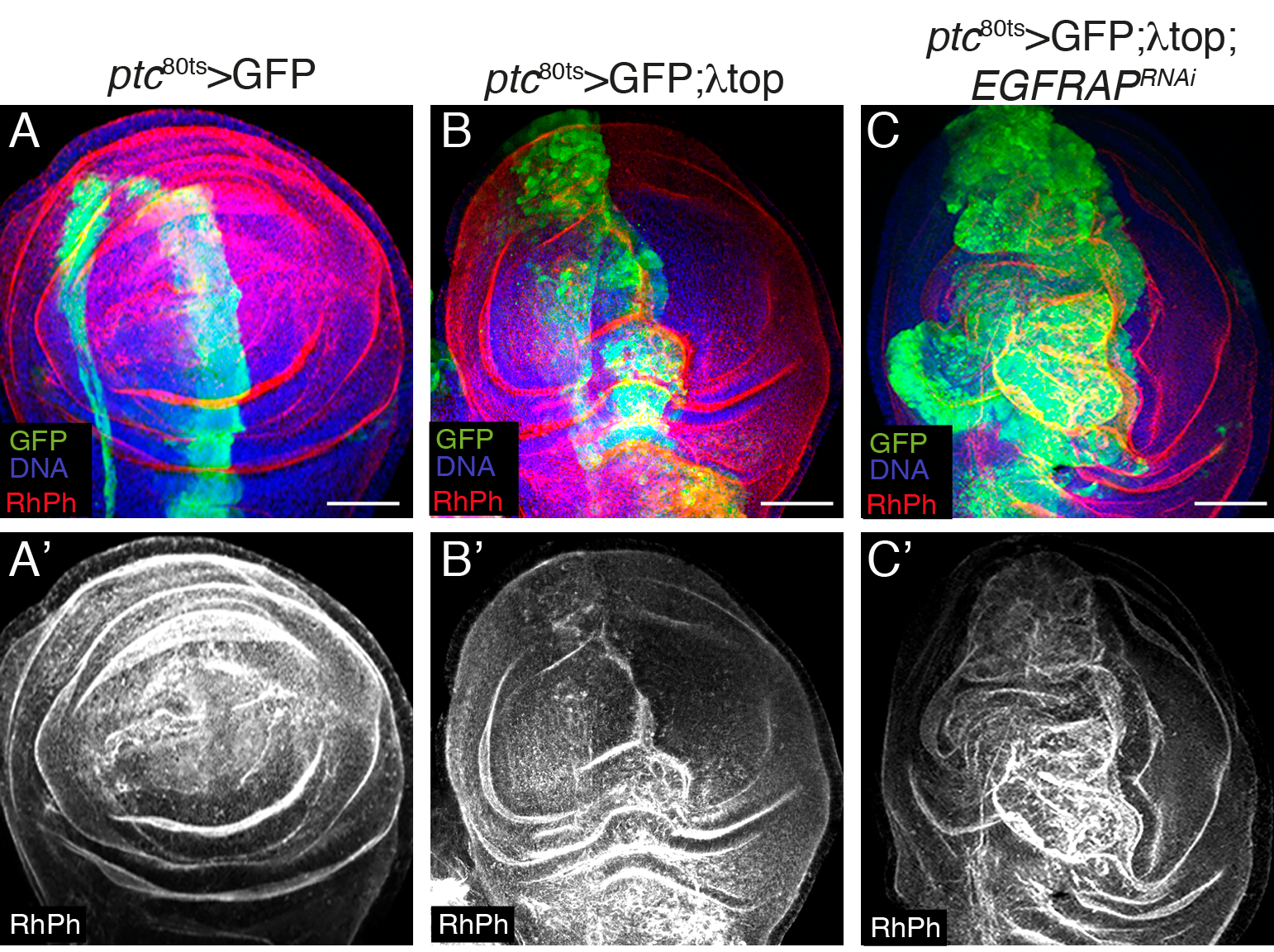

Supplement: S8 Fig — (A-C’) Maximal projection of confocal views of wing imaginal discs from third-instar larvae expressing the indicated UAS transgenes under the control of ptc80ts-Gal4 stained with anti-GFP (green), RhPh (red) and Hoechst (DNA, blue). Scale bars, 50 μm (A-C). (TIF) [file pgen.1009738.s008.tif]

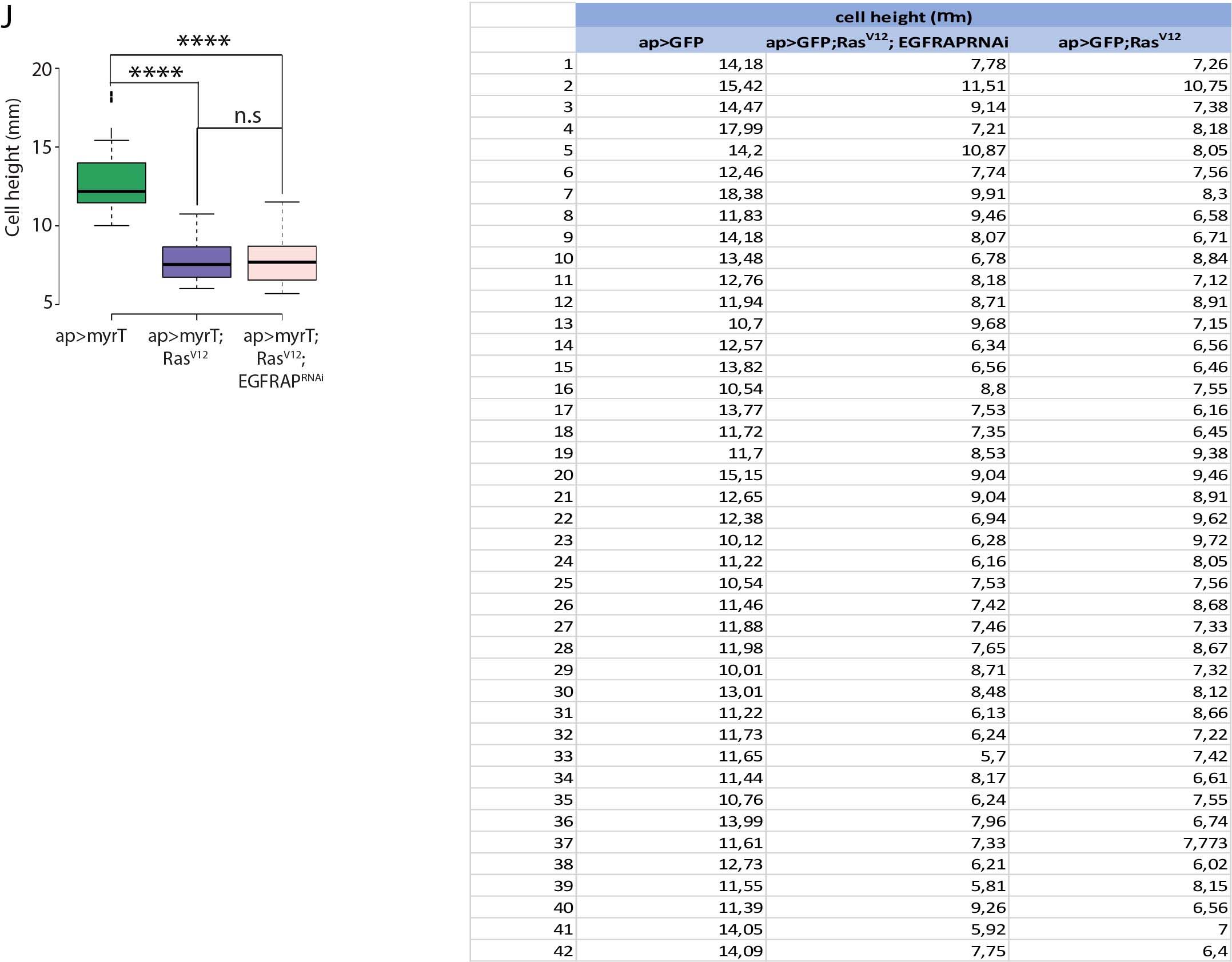

Supplement: S1 Data — File containing numerical raw data corresponding to Fig 2J. (JPG) [file pgen.1009738.s009.jpg]

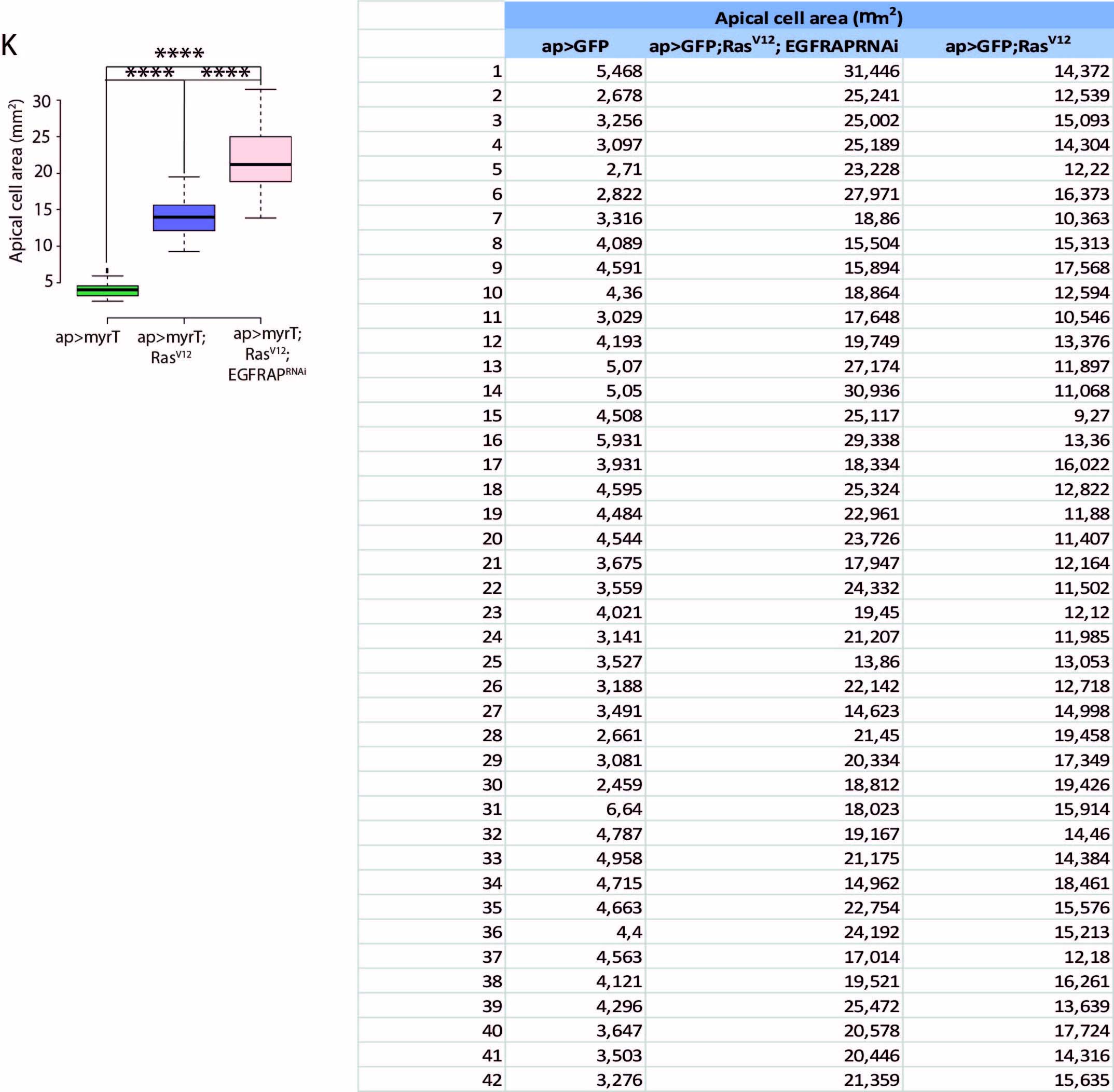

Supplement: S2 Data — File containing numerical raw data corresponding to Fig 2K. (JPG) [file pgen.1009738.s010.jpg]

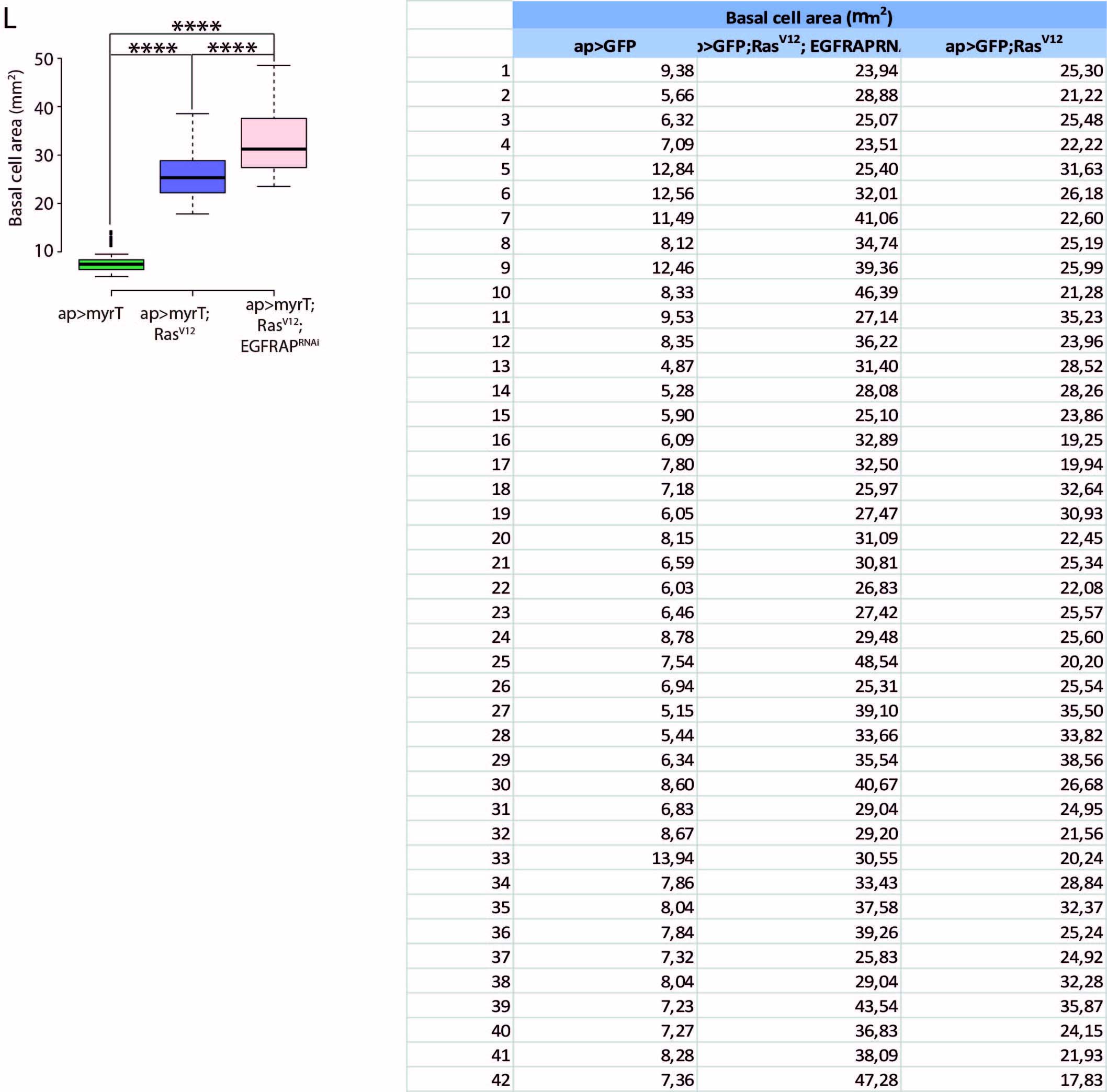

Supplement: S3 Data — File containing numerical raw data corresponding to Fig 2L. (JPG) [file pgen.1009738.s011.jpg]

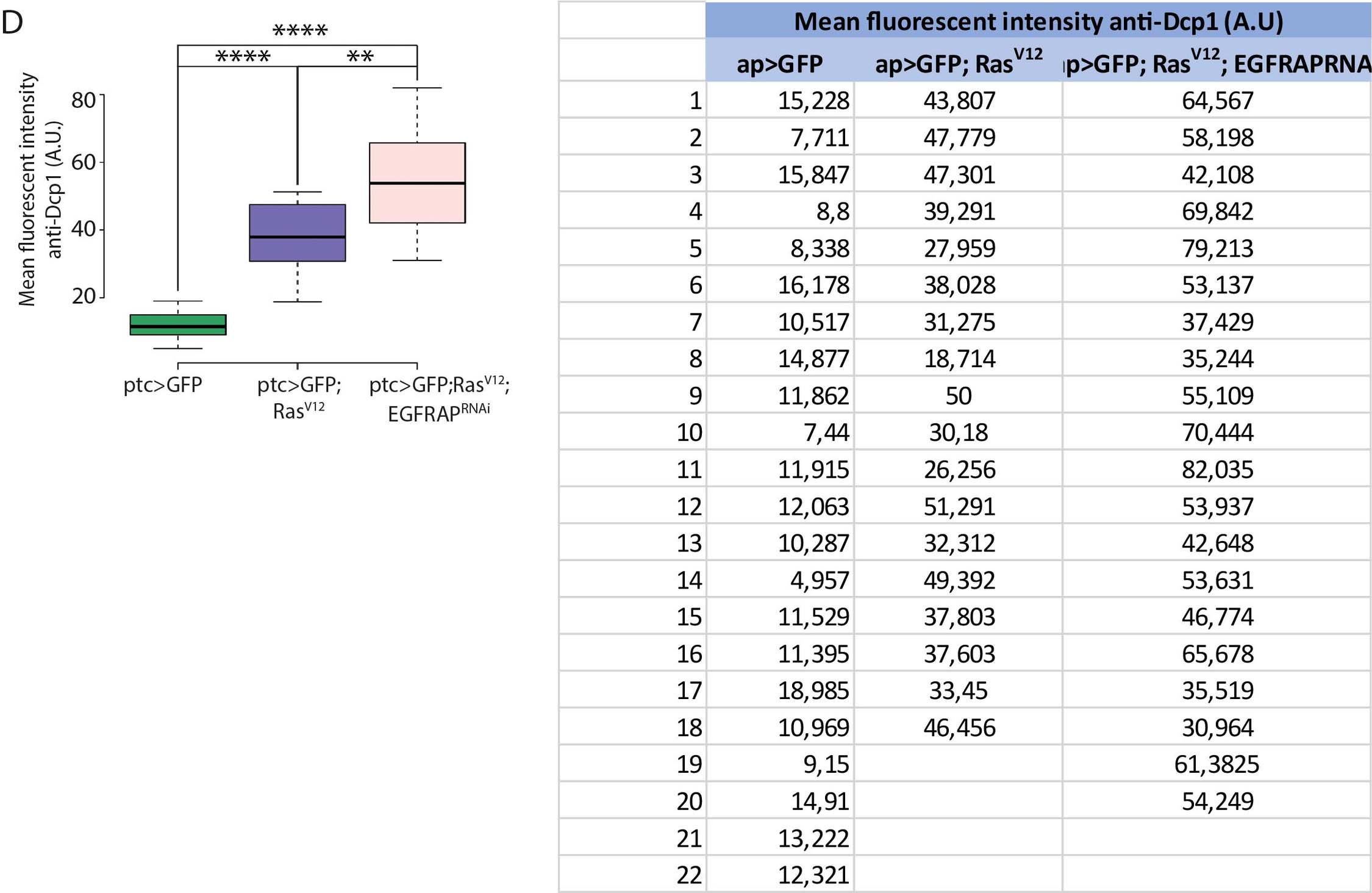

Supplement: S4 Data — File containing numerical raw data corresponding to Fig 3D. (JPG) [file pgen.1009738.s012.jpg]

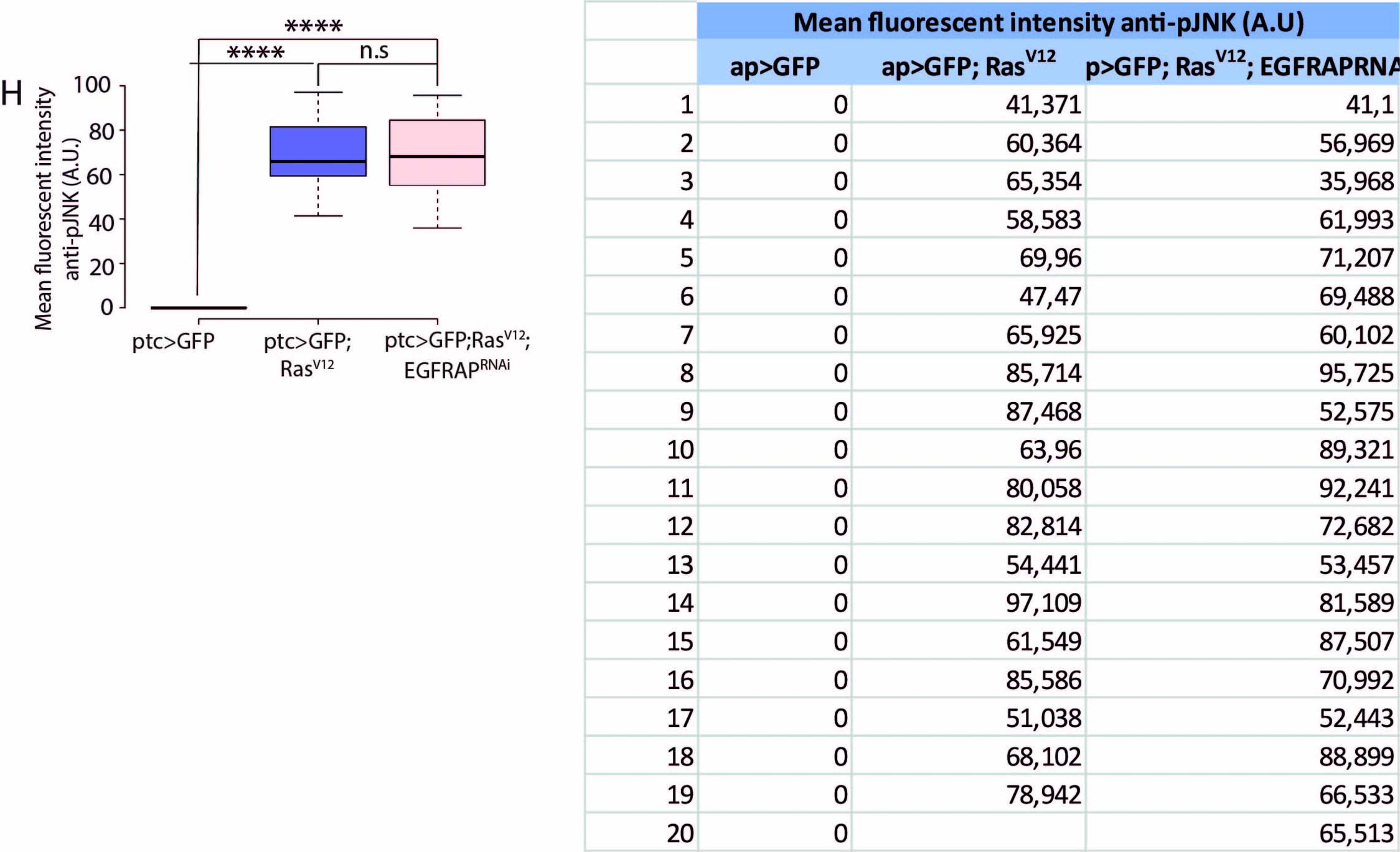

Supplement: S5 Data — File containing numerical raw data corresponding to Fig 3H. (JPG) [file pgen.1009738.s013.jpg]

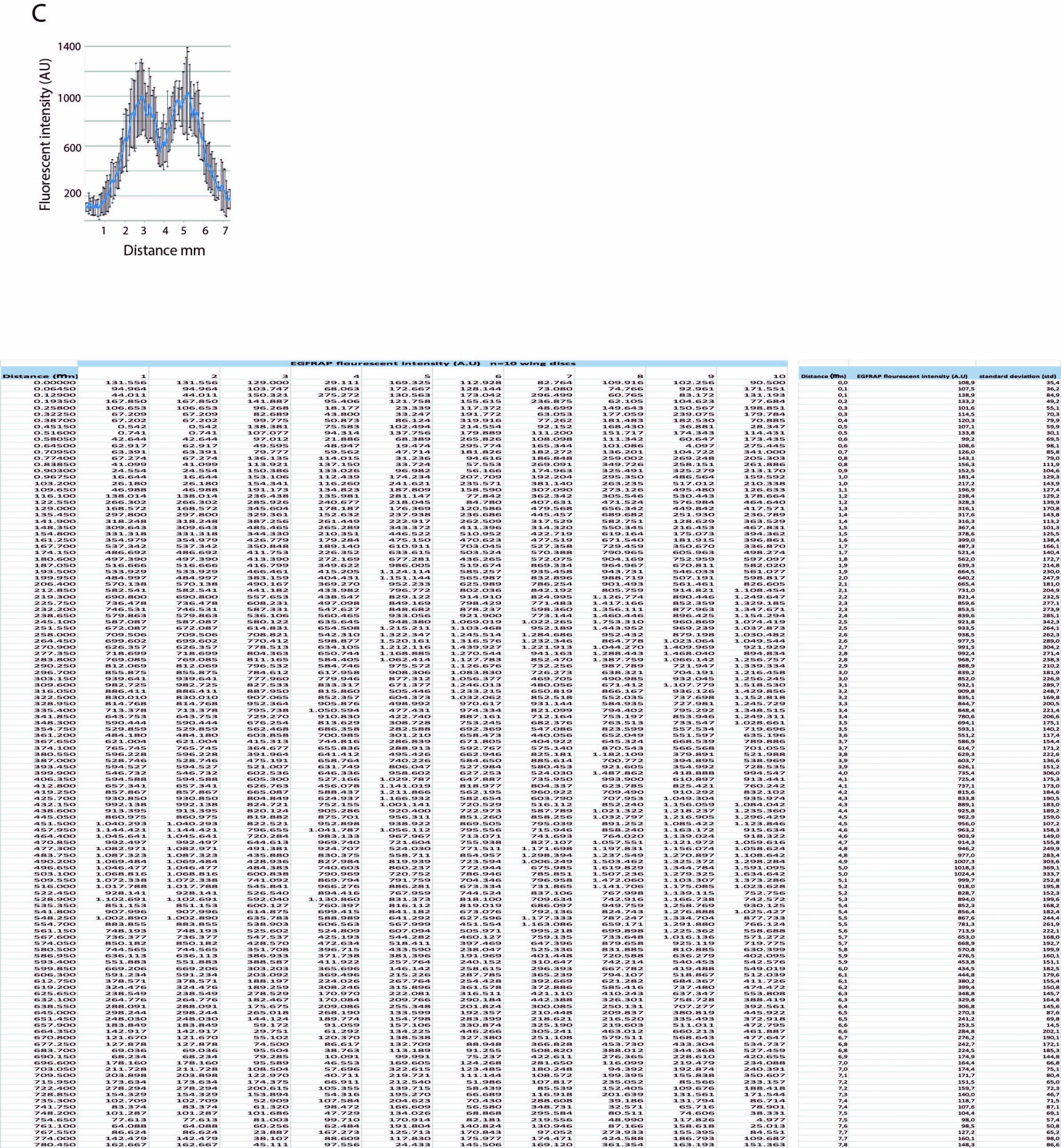

Supplement: S6 Data — File containing numerical raw data corresponding to Fig 6C. (JPG) [file pgen.1009738.s014.jpg]

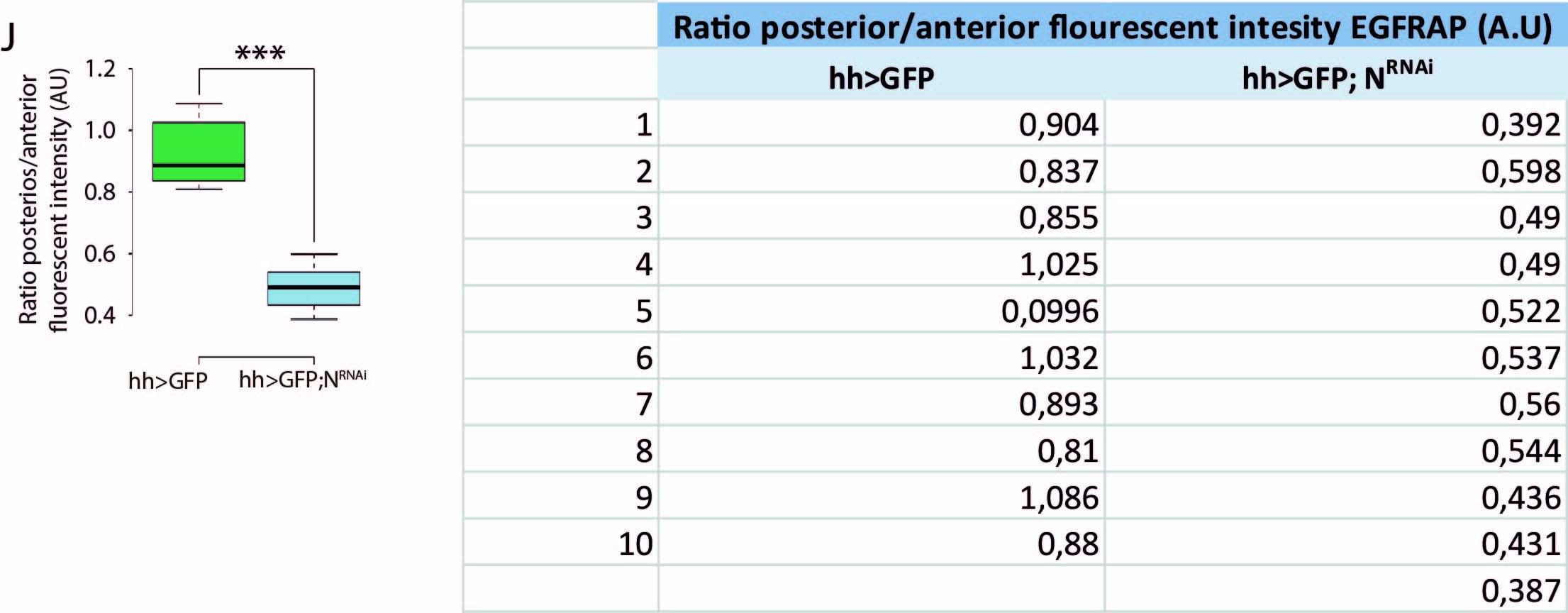

Supplement: S7 Data — File containing numerical raw data corresponding to Fig 6J. (JPG) [file pgen.1009738.s015.jpg]

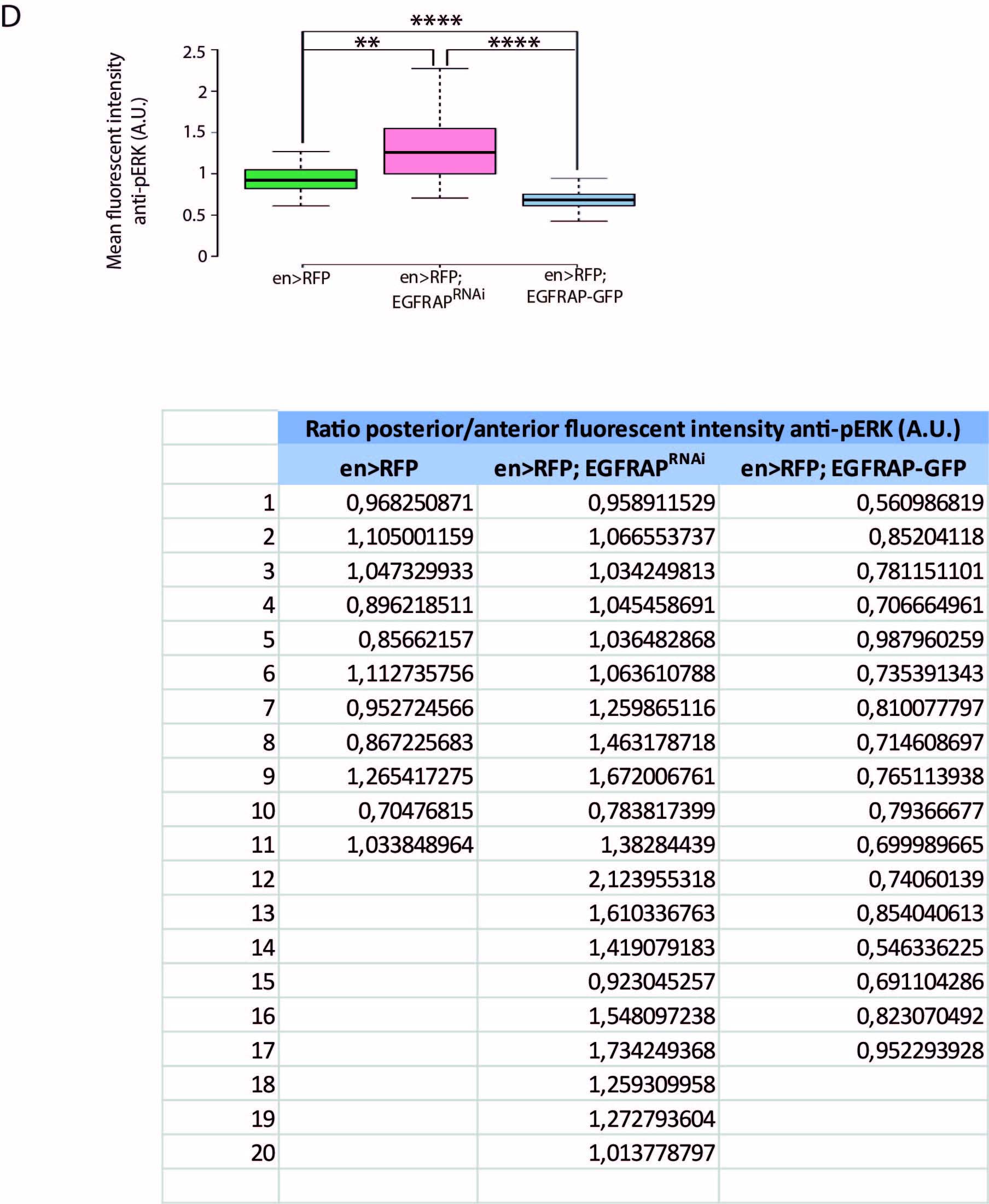

Supplement: S8 Data — File containing numerical raw data corresponding to Fig 7D. (JPG) [file pgen.1009738.s016.jpg]

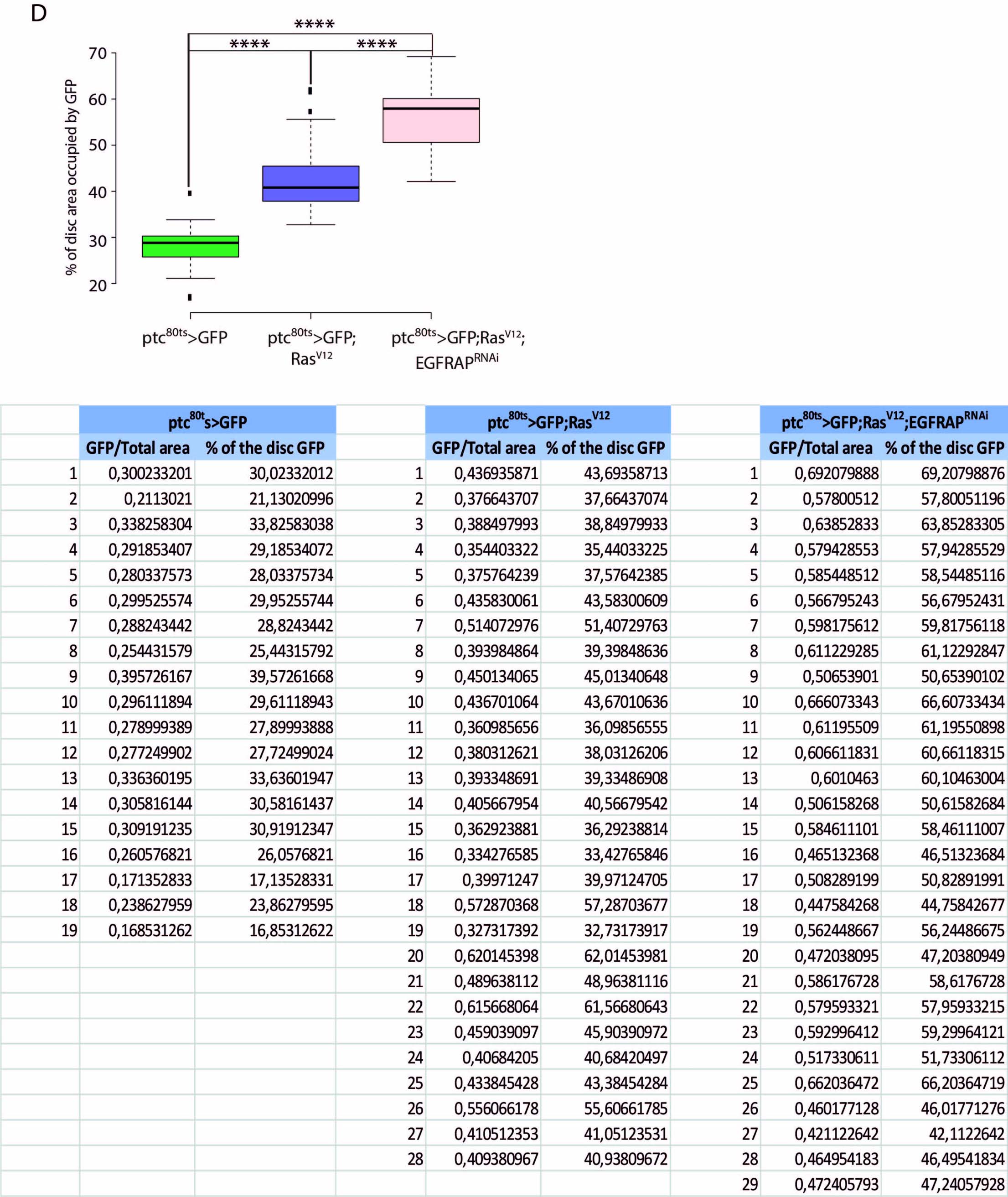

Supplement: S9 Data — File containing numerical raw data corresponding to S1D Fig. (JPG) [file pgen.1009738.s017.jpg]

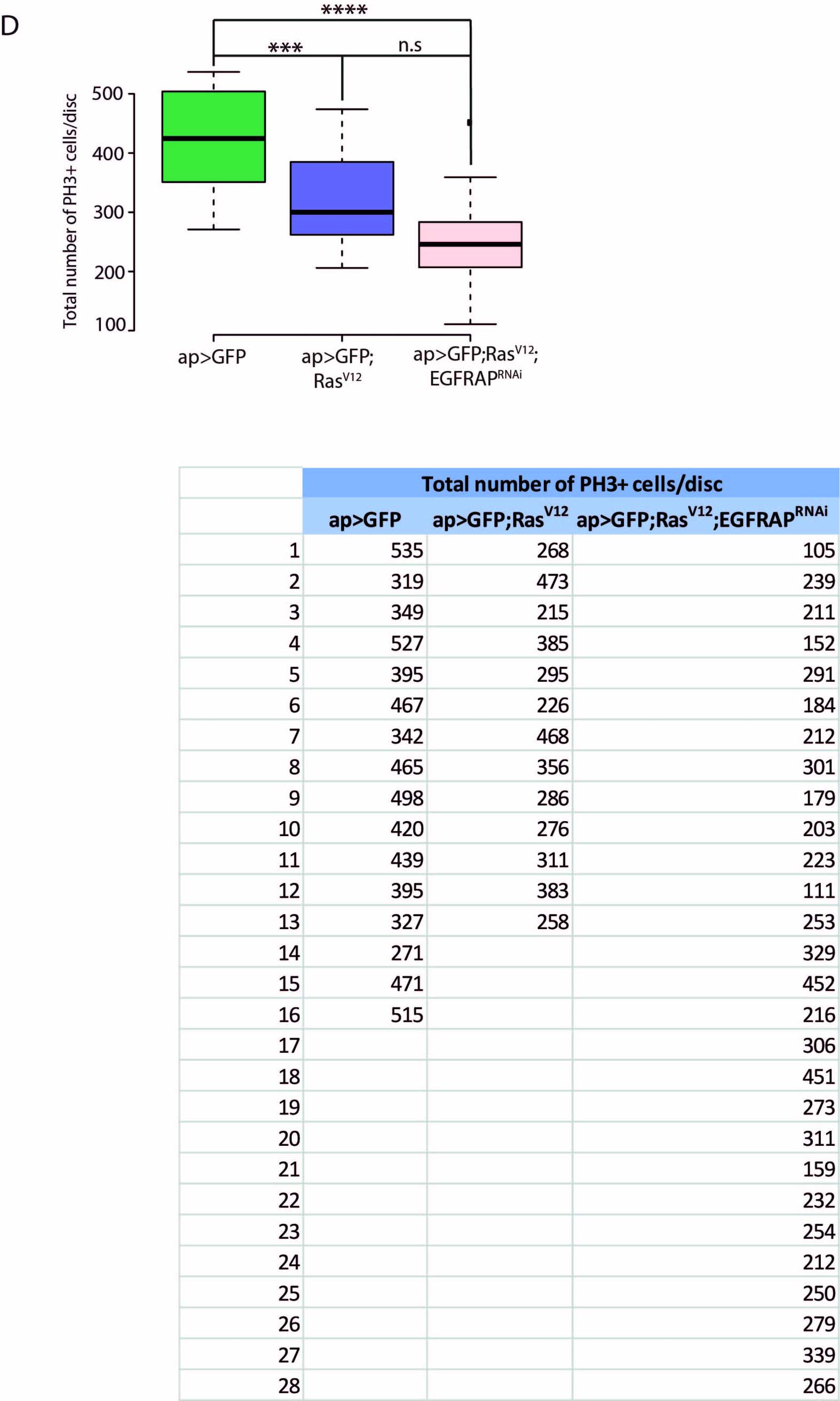

Supplement: S10 Data — File containing numerical raw data corresponding to S2D Fig. (JPG) [file pgen.1009738.s018.jpg]

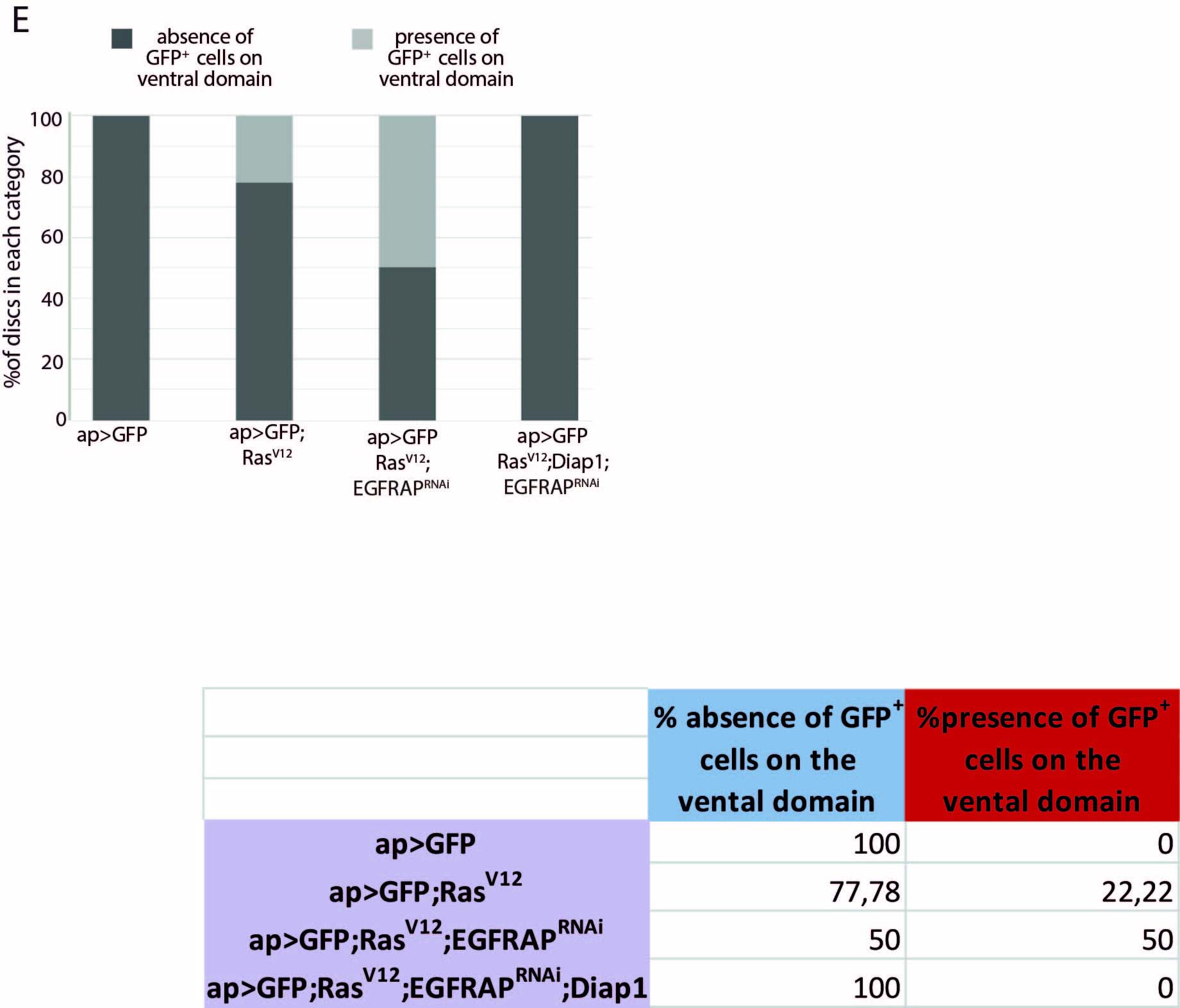

Supplement: S11 Data — File containing numerical raw data corresponding to S3E Fig. (JPG) [file pgen.1009738.s019.jpg]
