# Supplementary material for: EGFRAP encodes a new negative regulator of the EGFR acting in both normal and oncogenic EGFR/Ras-driven tissue morphogenesis
Source: PLoS Genet. 2021 Aug 19;17(8):e1009738. doi: 10.1371/journal.pgen.1009738 (PMC8407591; doi:10.1371/journal.pgen.1009738)
Supplement: Attachment — Submitted filename: Rebutal Letter Editor PLoS Genetics Final.docx [file pgen.1009738.s020.docx]

20th May 2021

**The Editor**

**PLoS Genetics**

MS ID#: PGENETICS-D-21-00125

MS TITLE: “**EGFRAP encodes a new SH2 domain-containing protein acting in a negative feedback loop that controls EGFR/Ras-driven tissue hyperplasia”**

Dear Editor,

Please find enclosed a detailed response to the Reviewers’ comments to our original submission PGENETICS-D-21-00125. Following your instructions, we have addressed most of the referees concerns and suggestions (see below), responses that have been added to the original submission. The revised MS contains three amended main Figures, one revised Supplementary Figure and two new Supplementary Figures.

The major changes in the MS are the following:

1. We have renamed the CG33993 protein as EGFRAP for EGFR Adaptor Protein (see response to Reviewer 2 minor comments below). The title and the text have been changed accordingly.

2. We have strengthened our findings demonstrating that EGFRAP behaves as an inducible inhibitor of the EGFR pathway. We now report that EGFRAP is also expressed in the eye disc. As we had shown for the wing disc, its expression in the eye disc is upregulated upon ectopic EGFR activation.

3. We now described that the normal *EGFRAP* expression pattern in the wing margin is regulated by the Notch pathway. We also show that the increase in EGFRAP levels observed in conditions of EGFR hyperactivation is due to the associated rise in Notch activity that occurs in this context. We have thus modified our model and now propose that, while *EGFRAP* behaves as a non-essential gene for normal morphogenesis, it becomes an important negative regulator of EGFR function in situations of excessive EGFR/Ras activity. EGFRAP specificity for cells with high EGFR activity is consistent with activation of a Notch pathway-mediated negative feedback loop, where it acts to help prevent excessive EGFR activity and uncontrolled cell growth.

4. We have made use of a GFP-EGFRAP fusion protein and a newly developed tool to visualize simultaneously endogenous EGFR protein and EGFRAP (Revatis et al. Dev.2020) to demonstrate that *EGFRAP* expression does not affect EGFR localization or levels, suggesting that *EGFRAP* could regulate EGFR activity downstream of receptor expression and localization.

5. We have cited in the Discussion additional examples of non-essential genes playing a key role in tumorigenesis to reinforce our proposition of the existence of buffering genetic mechanisms that are deployed only in the context of aberrant pathological signalling.

6. In our original submission we had reported that elimination of *EGFRAP* did not lead to increased EGFR activity. We now report the identification of an EGFRAP paralog PVRAP. Downregulation of *PVRAP* enhances the overgrowth and folding phenotypes due to the overexpression of Ras^V12^ alone or in combination with *EGFRAP RNAi*. Furthermore, while downregulation of either *EGFRAP* or *PVRAP* on their own does not cause any visible phenotype in the adult, we find that the simultaneous downregulation of both of them resulted in ectopic sensory organs in the notum, a phenotype that resembles increased EGFR activity. These results led us to propose that *EGFRAP* and *PVRAP* may act redundantly to downregulate EGFR activity in normal as well as in pathological conditions.

7. We have shortened the manuscript confining details to figures and keeping take-home messages in the text.

In summary, we are convinced the new results added to the MS increase the quality, interpretation and significance of our findings and provide stronger support for our conclusions. Thus, we would like to take this opportunity to thank the reviewers for their efforts and their very useful comments. We hope that you and the reviewers will agree that the new results improve the manuscript in a manner that you find it now suitable for publication in PLoS Genetics.

Yours sincerely

María Dolores Martín-Bermudo, PI.

**Reply to reviewers’ comments**

**Reviewer #1:** The paper by Soler Beatty describes a screen for inhibitors of activated EGFR/Ras signaling. Expression of activated Ras in the wing disc leads to deformed discs, and this phenotype was exacerbated by co-expression of CG33993 RNAi. The presence of an SH2 domain in CG33993 indicated a possible role in signaling. CG33993 null mutants display no phenotype. Normal expression of CG33993 is observed in some regions of EGFR activity but not in many others, and is upregulated following constitutive EGFR/Ras activity. Finally, over expression of CG33993 attenuates EGFR signaling.

Evaluating this paper is a close call, because the biological significance of CG33993 is unclear. Most of the conclusions were obtained under ectopic conditions. CG33993 RNAi phenotype was revealed only following expression of activated EGFR/Ras, while null mutants have no phenotype on their own. The normal expression pattern coincides only marginally with regions of prominent EGFR activation. It is however upregulated following constitutive EGFR/Ras activity, indicating some connection. The phenotype of over-expressed CG33993 could represent an indirect dominant-negative effect. Thus, it is clear that CG33993 has the capacity to attenuate EGFR/Ras signaling, possibly due to dominant-negative effects of its SH2 domain. However, whether it normally plays any role as an inducible inhibitor of the pathway is not convincingly demonstrated. I would recommend examining the normal expression pattern of CG33993 and expression after ectopic EGFR activation in additional tissues, such as the embryo or the eye disc, to solidify these findings which are the strongest aspect of the paper.

*We now show that CG33993, which we have renamed EGFRAP (see response to Reviewer 2 minor comments below), is also expressed in photoreceptor 7 in the eye disc and, as it is the case in the wing disc, this expression is upregulated upon EGFR overactivation.* Thus, EGFRAP expression and its relationship with EGFR during *Drosophila* eye development appears analogous to the situation in wing imaginal discs. *This data is shown in a new Supplementary Figure (Supplementary Fig.7).*

In the model (Fig. 8), it is not clear to me why the authors suggest that enhanced signaling downstream of the receptor, in the absence of CG33993, will lead to excess ligand processing. Rhomboid, the Spitz protease, is a downstream target of the pathway only in a very restricted set of tissues (eye disc and ovary).

*This was a proposal based on our findings that EGFRAP behaves as a modulator of oncogenic EGFR activity and results by others showing that oncogenic EGFR activity promotes tumor overgrowth in a feedback loop by stimulating expression of its ligand Spitz (Chabu et al. Nature Communications 2017). In this context, we envisioned that the increase in oncogenic EGFR activity due to EGFRAP elimination could result in a further increase in Spitz expression. However, we agree with the reviewer in that we have not proven this, so we have now removed this aspect in our model, which we have changed to accommodate new results showing regulation of EGFRAP by the Notch pathway (new version of Fig.8 and response to Reviewer2 and 3).*

Finally, I did not find the Figure Legends section.

*We are really sorry about this. The Figure Legends section has been added to the new version of the manuscript.*

**Reviewer #2**: In this paper, Beatty et al. identify the SH2-domain protein CG33993 as a novel feedback inhibitor of EGFR signaling. They show that knocking down CG33993 by RNAi enhances the wing disc overgrowth induced by expression of activated Ras. The effect seems to be largely due to increased cell growth and cell shape changes. Removing CG33993 or its SH2 domain by mutation has the same enhancing effect, but causes no phenotype in an otherwise wild-type background. CG33993 interacts with the intracellular domain of EGFR in a yeast two-hybrid assay, and mutating a series of tyrosines in EGFR diminishes this interaction. CG33993 is expressed adjacent to the DV boundary of the wing disc, in a domain of high EGFR activity, and the protein localizes apically within the cells. Increasing CG33993 levels reduces dpERK levels and alters wing morphology, and CG33993 levels are increased by EGFR activation.

These results are potentially interesting, as CG33993 homologues could act as tumor suppressors (although the authors do not discuss whether clear human homologues exist). However, the paper could be improved by addressing some additional points.

1) The authors do not show that normal CG33993 expression requires EGFR signaling. How is it affected in cells mutant for EGFR or a downstream component of the pathway? Why is it only expressed at the wing margin and not in the wing vein primordia?

*We have addressed this point and show that EGFR is not essential for the normal pattern of expression of CG33993, which we have renamed EGFRAP (see minor comments below). This is shown in a new version of Fig.6 (new Fig.6H). Instead, we find that the Notch pathway regulates EGFRAP expression in the wing margin region (new Fig.6I). We show that the ectopic expression of an intracellular active fragment of the Notch receptor (N^i^) results in ectopic EGFRAP expression (new Fig.6K). Furthermore, we find that the increase in EGFRAP levels observed upon EGFR pathway overactivation is a consequence of the rise in Notch activity occurring in these cells, as it can be blocked by co-expression of a Delta RNAi (new Fig.6L). These new results have led us to modify our model and propose that cells could minimize outcomes of excessive EGFR/Ras signalling by inducing the expression of the negative regulator of the pathway EGFRAP through activation of the Notch pathway (new version of Fig.8).*

2) The authors do not provide a mechanism by which CG33993 binding to the EGFR inhibits EGFR function. Is the level of EGFR protein or its intracellular localization altered by CG33993 overexpression?

*To address this point and analyse EGFR expression upon EGFRAP overexpression, we have used a recently developed tool, flies carrying a superfolder GFP inserted in the EGFR locus (EGFR-sfGFP) (Revaitis et al. Dev. 2020, doi: 10.1242/dev.183210). In addition, to be able to monitor EGFR-sfGFP and EGFRAP expression simultaneously in wing discs overexpressing EGFRAP, we have used flies, generated throughout the course of this study, carrying an untagged GFP version of EGFRAP, UAS-EGFRAP, which behaves as the previously used UAS-EGFRAP-GFP (see new version of Materials and Methods). Using these tools, we find that overexpression of EGFRAP (en>EGFRAP) did not affect the levels or localization of EGFR-sfGFP. This data is shown in a new modified version of Fig.7 (new Fig.7G-G’’’). These results suggest that the phenotypes associated to downregulation of EGFR signalling upon EGFRAP overexpression are not a consequence of defects in receptor localization or levels. These results lead us to propose that EGFRAP could regulate EGFR activity downstream of receptor expression and localization.*

3) It is not clear that the intracellular tyrosines in EGFR would undergo the appropriate phosphorylation in yeast. Can the authors show that mutating these to acidic residues maintains or increases the interaction, as predicted if it depends on phosphorylation?

*We agree with the reviewer that two hybrid experiments in yeast are not totally conclusive. However, this approach is commonly used as a first attempt to identify and map interactions between proteins in Drosophila. In addition, our results show that the interactions between EGFRAP and EGFR were stronger than those observed in the positive controls. Thus, we believe that our experiments and results are a good starting point to suggest that EGFRAP and EGFR interact. We think, and we hope the reviewer would understand, that generating a collection of mutants in Drosophila carrying the proposed mutations is beyond the scope of this work.*

Minor points:

1) A control disc with ap-GAL4 driving only CG33993 RNAi should be included in Fig. 1.

*This control has now been added in a new version of Fig.1 (new Fig.1C).*

2) In Fig. S2, PH3 would be better quantified as the number of labeled cells per disc rather than mean fluorescence intensity.

*Quantification has now been done according to the reviewer´s suggestion and added to a modified version of Sup.Fig.2D.*

3) It would much improve readability if the authors used the name they have chosen for their gene, loef, rather than CG33993, throughout the manuscript.

*We have now used the name chosen for CG33993 throughout the manuscript. However, due to new results showing a possible functional redundancy between CG33993 and its Drosophila paralog PVRAP, we have decided to rename the gene EGFRAP, for EGFR adaptor protein (see also response to Reviewer 3).*

4) The paper should be carefully proof-read. In addition to the lack of figure legends in the original submission, there are errors throughout. For instance, the numbers in the text do not match those in the graphs in Fig. 2K, L, it is unclear from the text and Fig. 5 whether CG33993 interacts with Btl or Htl, and when describing the generation of the CG33993 antibody, the authors state that region X-Y of the coding sequence was amplified by PCR.

*We are really sorry for this, we have now added Figure Legends to the text and carefully proof-read the manuscript.*

**Reviewer #3**: The manuscript describes characterization of CG33993, a Drosophila gene encoding a protein with an SH2 domain. Mutation or knockdown of CG33993 had no discernible phenotype in normal development. However, the gene was discovered in an RNAi screen as an enhancer of RasV12 phenotypes, suggesting the potential to inhibit pathway activity. A detailed examination in the wing disc showed RasV12 hyperplasia was enhanced by loss of function of CG33993 to exacerbate cell shape changes. CG33993 appears to be a target of the pathway and was induced when constitutively active EGFR was ectopically expressed. The connection to the EGFR pathway was further established through yeast-two-hybrid assays that showed interaction of CG33993 with certain residues in the EGFR intercellular domain known to interact with SH2 domain proteins. The authors conclude CG33993 participates in a negative feedback loop and suggest it would have the potential to modify EGFR/Ras activity in cases of elevated signaling, even though a role in normal physiology was not observed.

The work is of a high standard and represents a thorough characterization of the gene including expression analysis to the level of the sub-cellular localization of the protein. Genetic evidence that CG33993 can inhibit EGFR/Ras is convincing. The yeast-two-hybrid results are a good starting point towards defining the molecular mechanism whereby CG33993 could bind and inhibit EGFR. However, the description of the various mutants was quite confusing. The details could be confined to the figure and the take-home lesson kept in the text.

*We have now removed the description of the various mutants from the main text.*

Given that an antibody is in hand for both proteins (tagged reagents could also be used), it should be possible to demonstrate this interaction is physiological by IP experiments in Drosophila cells and this would provide stronger evidence.

*We agree with the reviewer that IP experiments in Drosophila cells would provide stronger evidence for a physiological interaction between EGFR and CG33993. Unfortunately, CG33993, which we have now renamed EGFRAP, is not expressed in S2 cells (Flybase) and only at low levels in a limited number of wing disc cells. In this manner, the IP experiments will require ectopic expression of EGFRAP, and most likely EGFR, in S2 cells, which could also result in interactions that might not reflect physiological conditions. We agree with the reviewer that the yeast-two-hybrid results can only be used as a good starting point to suggest interactions between EGFRAP and EGFR interact, and this is what we state in the manuscript.*

The idea that genetic mechanisms exist only to be deployed in aberrant signaling situations is an interesting one and the authors cite some examples. However, the overall case of the importance of studying CG33993 to understand the role of Ras in tumorigenesis fell short of being convincing. A more robust discussion of why non-essential genes are interesting and citing more relevant literature could bolster the case. The closing remarks about finding more genes like CG33993 for their value as therapeutic targets in cancer was weak. Rather than increasing enthusiasm for the project, this emphasized that this detailed study of CG33993 had not cast much light on its own potential in this regard.

*We have now extended the discussion of why non-essential genes are interesting in the context of aberrant signalling by providing more examples and appropriate literature.*

Related to the above point, redundancy could be masking a phenotype in the mutant. Very little discussion of the molecular structure of the CG33993 was provided, but there was some mention of the related Tensin1-3 genes. Are there any genetic interactions with these genes? Moreover, the authors cite the correlation of Tensin1-3 loss in cancer, which prompts the question of what about CG33993? I think it may not be easy to recognize mammalian homologs if there are no domains in addition to the SH2 domain, but a fuller discussion of this is needed, or the reader is left with questions about the molecular nature of the gene and whether it could be relevant to cancer.

*The reviewer is right in that the absence of a loss-of-function phenotype for EGFRAP could be due to compensation by closely related proteins. In fact, we noticed that adjacent to EGFRAP lies the gene PVRAP, which also contains a SH2 domain and is considered to be a paralog of EGFRAP (FlyBase). Furthermore, the amino acid sequences of their SH2 domains share 74% identity, versus 26%-35% identity for pairwise comparisons of EGFRAP with other Drosophila SH2 domain containing proteins. We have found that, as it is the case for EGFRAP, expression of an RNAi against PVRAP in wing imaginal discs enhanced the Ras^V12^ overgrowth and folding phenotype. This data has been incorporated in a new Supplementary Figure (new Sup.Fig.6). Furthermore, downregulation of PVRAP enhanced the effects of eliminating EGFRAP in Ras^V12^ expressing wing discs (new Sup.Fig.6B-D’). In addition, while downregulation of either EGFRAP or PVRAP on their own did not cause any visible phenotype in the adult (new Sup.Fig.6F-G, I-K), the simultaneous downregulation of both of them resulted in ectopic sensory organs in the notum (new Sup.Fig.6H), a phenotype that resembles increased EGFR activity, and blisters in the wing (new Sup.Fig.6L). These results led us to propose that EGFRAP and PVRAP may act redundantly to downregulate EGFR activity in normal as well as pathological conditions. These new results have been added to the manuscript.*

The manuscript needs attention. For example, there is a repeated sentence in the abstract, references are not fully formatted, there were no figure legends (that I could find) and throughout the text would benefit from consultation with a copy editor. Reducing the length of the manuscript (by as much as half) would improve impact and readability.

*We are really sorry for these errors. We have now carefully proof-read and shorten the manuscript.*

Small point: The authors suggest a name for the gene but do not use it.

*We have now used the name EGFRAP throughout the text.*
