# Supplementary material for: EGFRAP encodes a new negative regulator of the EGFR acting in both normal and oncogenic EGFR/Ras-driven tissue morphogenesis
Source: PLoS Genet. 2021 Aug 19;17(8):e1009738. doi: 10.1371/journal.pgen.1009738 (PMC8407591; doi:10.1371/journal.pgen.1009738)
Supplement: Attachment — Submitted filename: Rebutal Letter Editor PLoS Genetics 2nd Revision.docx [file pgen.1009738.s021.docx]

01st July 2021

**The Editor**

**PLoS Genetics**

MS ID#: PGENETICS-D-21-00125R

MS TITLE: “***EGFRAP encodes a new negative regulator of the EGFR acting in both normal and oncogenic EGFR/Ras-driven tissue morphogenesis*”**

Dear Editor,

Please find enclosed a detailed response to the referees’ comments to our revised submission PGENETICS-D-21-00125R. Following your instructions, we have addressed the specific points made by each reviewer (see below), which have been added to the revised submission. The revised MS contains 2 main amended Figures (Fig.5 and 8), and one revised Supplementary Figure (Sup. Fig. 6).

The major changes of the MS are:

1. We have moved the figure showing results from the Y-2-H from main figures to a Supplementary Figure, Sup.Fig.6.

2. We have moved the figure showing redundancy between EGFRAP and PVRAP from supplementary to main Figures (new Figure 5).

3. We have changed the title to accommodate the role of EGFRAP in morphogenesis. The new title is: “***EGFRAP encodes a new negative regulator of the EGFR acting in both normal and oncogenic EGFR/Ras-driven tissue morphogenesis*”.**

4. We have changed the discussion and incorporated a new section, “The function of EGFRAP in morphogenesis and oncogenic transformation”, where we discuss the function of EGFRAP, as a way to enhance robustness of the EGFR pathway, and place our results in the context of the proposal of the existence of an optimal level of Ras activation to elicit transformation, as suggested by Reviewer 3.

5. Finally, we have changed the model to accommodate the redundant function of EGFRAP and PVRAP during both morphogenesis and oncogenic transformation.

We believe the changes made to the manuscript improve the interpretation and significance of our findings. We would like to take this opportunity to thank all three reviewers for the efforts and very useful comments. We hope that you and the reviewers will agree that the new results improve the manuscript in a manner suitable for publication in PLoS Genetics.

Yours sincerely

María Dolores Martín-Bermudo, PI

**Reply to reviewers’ comments**

**Reviewer #1**: By addressing the reviewers comments, the authors have significantly improved the paper. It is now presenting the findings from a more balanced angle that is appropriate for publication.

Regarding the point raised by Reviewer 2 about the credibility of the 2-hybrid assay for SH2/P-Tyr binding, I did not find the author's comments convincing. Instead of placing this finding as a proof for EGFRAP/EGFR association, it would be better to say after the initial identification of EGFRAP in the activated Ras screen, that previously a 2-hybrid assay has also identified an interaction between the two proteins (Supp material), and not use it as an assay for structure-function studies of the interaction between EGFRAP and EGFR.

*We have moved the figure showing results from the Y-2-H from main figures to a Supplementary Figure, Sup.Fig.6. However, instead of placing these results after the initial identification of EGFRAP in the activated Ras screen, as suggested by the reviewer, we have incorporated them in the section “EGFRAP is expressed in wing margin cells and co-localizes apically with EGFR”, after the results showing co-localization of EGFRAP with EGFR-sfGFP, where we believe it fits better.*

**Reviewer #2:** The authors have made a good effort to address the points raised by all the reviewers, and have significantly improved the manuscript.

**Reviewer #3:** In this revised version, the authors have responded to the previous review. This led to additional experiments, which have quite radically changed the understanding of the biological role and control of EGFRAP. While this is progress, it is worrying that the previous version, if it had been published, would instantly have been in need of extensive revision. The new findings call for a different ‘angle’ on the results. EGFRAP appears to act redundantly with PVRAP as a negative regulator of EGFR. The redundant function of these genes therefore provides buffering and robustness in normal physiology. And that is quite different than a role that becomes evident only in an oncogenic background—so the title does not seem to fit the current version. This is a major concern.

*In the first version of our work, we proposed that EGFRAP had a limited capacity to negatively regulate EGFR during normal morphogenesis, yet behaved as an important regulator of cellular overgrowth driven by excessive EGFR/Ras signalling. We agree with the reviewer in that our findings showing that EGFRAP could act redundantly with PVRAP have changed our understanding of EGFRAP function during normal morphogenesis and that this is an important point. We also agree with the reviewer in that this new result points to a function of EGFRAP that is not exclusive of an oncogenic context. We tried to bring this point in the first revised version both in the results and discussion, where we incorporated in the “EGFRAP tumor cell specificity” section a paragraph commenting on the possible functional redundancy between EGFRAP and PVRAP to regulate EGFR activity*. *However, it is clear this has not been sufficient to pinpoint our latest view of EGFRAP function during morphogenesis. Thus, in this new revised version of the article, and following the advice of the reviewer, we have made the following changes to highlight this new aspect:*

*1. We have changed the title to accommodate the role of EGFRAP in morphogenesis. The new title is:* “***EGFRAP encodes a new negative regulator of the EGFR acting in both normal and oncogenic EGFR/Ras-driven tissue morphogenesis*”.**

*2. We have moved the figure showing redundancy between EGFRAP and PVRAP from supplementary to a main Figure, Figure 5 (see below).*

*3. We have changed the abstract to highlight the redundant function of EGFRAP in normal physiology from the beginning.*

*4. We have also changed the discussion and incorporated a new section, “The function of EGFRAP in morphogenesis and oncogenic transformation”, where we discuss the redundant function of EGFRAP, as a way to enhance robustness of the EGFR pathway. In addition, we suggest that the ability of EGFRAP to functionally inhibit EGFR activity in oncogenic cells on its own might arise from the increase in EGRAP expression that occurs in these cells. This is supported by our results showing that overexpression of EGFRAP in normal cells results in phenotypes associated to reduction of EGFR function.*

Other points:

1) The Y-2-H experiment does not warrant a main figure and should be a supplement, as the authors note it is just a beginning step. Even if EGFRAP and EGFR would have to be overexpressed in S2 cells—this is still a superior experiment and an S2 line expressing EGFR already exists.

*This point has also been raised by Reviewer 1. Thus, we have now deleted the section of the Y-2-H results “EGFRAP associates with activated EGFR” and incorporated a summarized version of these results into the section “EGFRAP is expressed in wing margin cells and co-localizes apically with EGFR”, after the results showing co-localization of EGFRAP with EGFR-sfGFP. We have also moved the figure showing results from the Y-2-H to a Supplementary Figure, Sup.Fig.6.*

2) In contrast to the Y-2-H experiment, the redundancy with PVRAP is an important result and should be a main figure. This type of redundancy, in neighboring genes has many precedents and as described above explains the lack of a phenotype in either gene alone.

*We have followed the suggestion by the reviewer and we have moved the figure showing redundancy between EGFRAP and PVRAP from supplementary to a main Figure, Fig.5.*

3) The discussion relative to human cancer still appears underdeveloped. For example, the second sentence in the abstract—' However, activated Ras alone is insufficient to produce malignancy.’ The multi-hit nature of cancer is well known and activation of Ras in the absence of other genetic alterations causes oncogene-induced senescence and indeed there may be an optimal level of Ras activation for tumorigenesis (comment on recent study in eLife: <https://elifesciences.org/articles/69192> ). EGFRAP and PVRAP appear to a case of two related genes that together have tumor suppressor function.

*We thank the reviewer for the suggestion of discussing our results in the context of the proposal of the existence of an optimal level of Ras activation for oncogenesis. We have now tried to incorporate this aspect in a new paragraph in the discussion:*

“EGFR autocrine loop is required for transformation by activated Ras in different mammalian cell systems, including fibroblasts, keratinocytes, intestinal epithelial cells, melanomas and pancreatic cells [21-23]. The contribution of EGFR to oncogenic transformation seems to be evolutionary conserved, as it has also been found in *Drosophila* [30] and in Xiphophorus fish [63]. Furthermore, EGFR stimulates Ras-dependent tumor overgrowth through canonical EGFR signaling. Thus, even though oncogenic Ras isoforms are constitutively active, EGFR activation can induce GTP loading on wild type Ras isoforms, enhancing effector pathway signaling [64]. In this context, recent studies in mice indicate the existence of an optimal level of Ras activation, the so-called “sweet spot”, conducive to tumor formation and progression (reviewed in [65]. Thus, any negative regulator of the EGFR, such as EGFRAP and PVRAP, could act as tumor suppressors of the overgrowth of wild type cells ectopically expressing Ras^V12^. Elimination of EGFRAP and/or PVRAP in these cells would lead to hyperactivation of the EGFR, which, in turn, would result in an enhancement of endogenous wild-type Ras signaling, thus increasing pathway activity and overgrowth. Alternatively, or in addition, the increase in EGFR activity due to elimination of EGFRAP and/or PVRAP could induce further overgrowth of Ras^V12^ cells by stimulating non-canonical EGFR pathways. In fact, the EGFR stimulates the growth of fly and human cancer cells harboring oncogenic Ras mutations through the activation of pathways controlling cell growth and survival, such as the Hedgehog pathway [24].”

**Have all data underlying the figures and results presented in the manuscript been provided?**

Large-scale datasets should be made available via a public repository as described in the *PLOS Genetics* [data availability policy](http://journals.plos.org/plosgenetics/s/data-availability), and numerical data that underlies graphs or summary statistics should be provided in spreadsheet form as supporting information.

Reviewer #1: Yes

Reviewer #2: **No:** The numerical data underlying the graphs presented are not included.

*We have now included all numerical data underlying the graphs.*

Reviewer #3: Yes
